# Rab6 is required for rapid, cisternal-specific, intra-Golgi cargo transport

**DOI:** 10.1038/s41598-020-73276-w

**Published:** 2020-10-06

**Authors:** Lindsey James Dickson, Shijie Liu, Brian Storrie

**Affiliations:** Department of Physiology and Biophysics, University of Arkansas for Medical Sciences, Little Rock, AR 72205 USA

**Keywords:** Membrane trafficking, Golgi, Small GTPases

## Abstract

Rab6, the most abundant Golgi associated small GTPase, consists of 2 equally common isoforms, Rab6A and Rab6A′, that differ in 3 amino acids and localize to trans Golgi cisternae. The two isoforms are largely redundant in function and hence are often referred to generically as Rab6. Rab6 loss-of-function inhibits retrograde Golgi trafficking, induces an increase in Golgi cisternal number in HeLa cells and delays the cell surface appearance of the anterograde cargo protein, VSVG. We hypothesized that these effects are linked and might be explained by a cisternal-specific delay in cargo transport. In pulse chase experiments using a deconvolved, confocal line scanning approach to score the distribution of the tsO45 mutant of VSVG protein in Rab6 depleted cells, we found that anterograde transport at 32 °C, permissive conditions, through the Golgi apparatus was locally delayed, almost tenfold, between medial and trans Golgi cisterna. Cis to medial transport was nearly normal as was trans Golgi to TGN transport. TGN exit was unaffected by Rab6 depletion. These effects were the same with either of two siRNAs. Similar intra-Golgi transport delays were seen at 37 °C with RUSH VSVG or a RUSH GPI-anchored construct using a biotin pulse to release the marker proteins from the ER. Using 3D-SIM, a super resolution approach, we found that RUSH VSVG transport was delayed pre-trans Golgi. These visual approaches suggest a selective slowing of anterograde transport relative to 3 different marker proteins downstream of the trans Golgi. Using a biochemical approach, we found that the onset of VSVG endoglycosidase H resistance in Rab6 depleted cells was delayed. Depletion of neither Rab6A or Rab6A′ isoforms alone had any effect on anterograde transport through the Golgi suggesting that Rab6A and Rab6A′ act coordinately. Delayed cargo transport conditions correlate strongly with a proliferation of Golgi cisternae observed in earlier electron microscopy. Our results strongly indicate that Rab6 is selectively required for rapid anterograde transport from the medial to trans Golgi. We suggest that the observed correlation with localized cisternal proliferation fits best with a cisternal progression model of Golgi function.

## Introduction

Rab proteins, the most numerous of the small GTPases that comprise the Ras superfamily in mammals, act as binary, molecular switches that in the GTP-bound state recruit effector proteins. In the case of Ras, the original example small GTPase, effector recruitment modulates cell signaling pathways such as the Ras/mitogen activated protein kinase (MAPK) pathway that is essential to the regulation of cell cycle, differentiation, growth, cell senescence and apoptosis. In the case of Rab proteins, effector recruitment variously modulates intracellular vesicular trafficking at the level of vesicle formation and budding, transport, and vesicle fusion and cargo release into the acceptor compartment^1^. In brief, the phenotypic consequences of any small GTPase depend on the individual protein and its effector interactions.

For the Golgi apparatus, the central organelle within the secretory pathway, roughly 20 different Rab proteins have been identified that affect Golgi organization in one way or another (for recent reviews, see^2–4^). Of these, Rab6 is the most abundant Golgi Rab^5^ and the example protein for the Rab6 subfamily^6,7^. The two Rab6 isoforms, Rab6A and Rab6A′ are alternate splicing forms that are expressed in equal amounts and differ by 3 amino acids in sequence. Collectively, we refer to Rab6A and Rab6A′ as Rab6. Likely, much of their function is redundant with the only known effector difference being the preferential binding of Kif20A (aka Rabkinesin-6/MKlp2) by Rab6A^7^. Both localize similarly to the trans-Golgi^8^. In total, Rab6 interacts with 15–20 different effectors and mediates multiple Golgi associated trafficking steps that affect both anterograde and retrograde Golgi vesicle trafficking^9^. Utskarpen et al.^10^ found that both Rab6A and A′ are involved in the transport of ricin from endosomes to the Golgi apparatus. Rab6A knockdown inhibits ricin transport to the TGN of Golgi apparatus, an inhibition that can be rescued by up-regulation of Rab6A′, a strong indication that Rab6A and A′ have overlapping roles in retrograde trafficking^10,11^. Similarly, using a RNAi approach, Young et al.^12^ found that depletion of either Rab6A or A′ delayed Golgi-resident protein recycling to the ER. Moreover, overexpression of GTP-locked form of either Rab6a or a′ stimulates Golgi-to-ER recycling of Golgi resident glycosylation enzymes^12–14^.

Perhaps, surprisingly considering the wide range of Rab6 effectors, Rab6 gain- or loss-of-function has strikingly different, nearly binary effects on overall Golgi organization. Expression of GTP-locked Rab6 or even overexpression of wild type Rab6 results in Golgi proteins redistributing from the juxtanuclear Golgi ribbon to the endoplasmic reticulum (ER)^13–15^. In essence, retrograde trafficking predominates, perhaps due to reinforced interactions with low affinity effector(s). In contrast, Rab6 knockdowns in culture or Rab6 knockout in mice result in little to no change in Golgi organization as assayed by the light microscope^12,14,16,17^. At first glance, Rab6 gain-of-function is more important to Golgi organization than loss-of-function is. However, when examined in more detail, Rab6 loss-of-function does have definite phenotypic consequences, for example, inhibition of actomyosin-dependent fission of transport vesicles at the Golgi apparatus^9^, delayed cell surface appearance of anterograde cargo^17–19^, and most significantly Rab6 knockout in mice is embryonically lethal indicating that Rab6 is essential at the organismic level^17^. At the electron microscope level, increased cisternal continuity and pronounced accumulation of budding and free vesicles in association with the trans Golgi/TGN has been observed across cell systems^17,18,20^. In sum, these experimental outcomes suggest a direct, localized involvement of Rab6 in Golgi vesicle trafficking. Consistent with this in HeLa cells^18,21^. Rab6 loss-of-function results in increased cisternal number suggestive of retrograde trafficking effects having a preferential effect on cisternal progression.

In this study, we have investigated the hypothesis that Rab6 regulates in a local, cisternal-specific manner, intra-Golgi, anterograde cargo transport. As expected, our line scan fluorescence mapping approaches placed Rab6 at the trans-Golgi with little to no co-distribution with medial or TGN markers. Rab6 loss-of-function supported normal transport of the temperature sensitive VSVG mutant protein, tsO45-G, from the ER to the medial Golgi and normal exit kinetics at the TGN at 32 °C, permissive conditions. However, cargo transport kinetics between the medial Golgi and trans Golgi were strongly inhibited. Furthermore, a similar strong intra-Golgi delay in anterograde transport was observed with two different, biotin-responsive, RUSH constructs at 37 °C with Rab6 knockdown but not individual Rab6a or Rab6a′ knockdowns, conditions in which Golgi cisternal number remains normal^21^. However, when co-depleted, Rab6a and Rab6a′ loss-of-function resulted in delayed cargo transport, an event that correlates strongly with the previously observed increase in cisternal number^21^. We conclude that Rab6a and Rab6a′ act cooperatively to regulate Golgi structure/function relationships in a cisternal-specific manner and in so doing provide the first evidence for a cooperative, functional interaction between two Rab isoforms. We suggest that the strong correlation with localized cisternal proliferation observed is best explained by a cisternal maturation/progression model of Golgi function.

## Results

### Rab6 is required for the rapid, intra-Golgi transport of tsO45G protein

**Figure 1:**
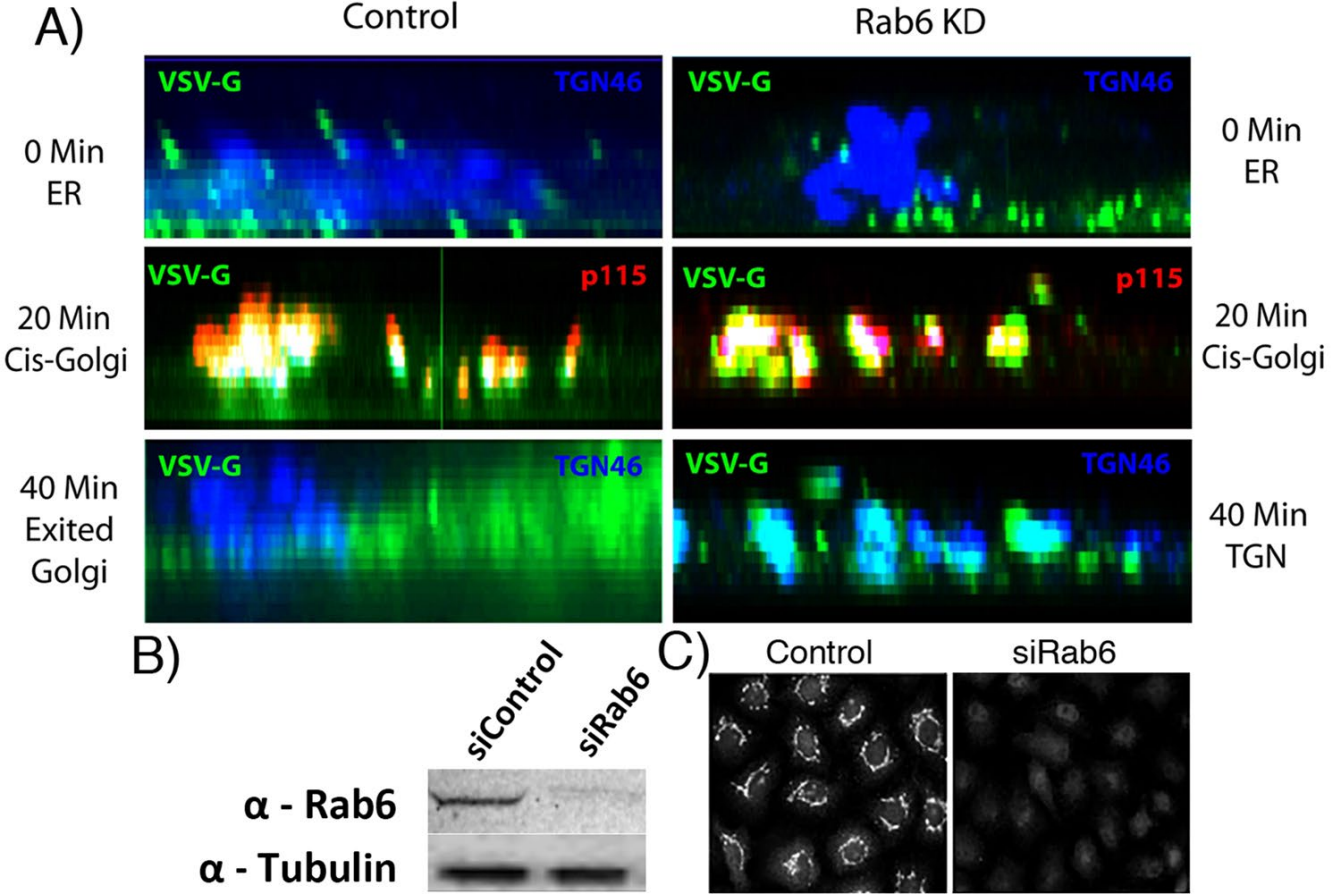
Rab6 knockdown strongly inhibits intra-Golgi cargo transport. Confocal immunofluorescence, orthogonal views of tsO45G-GFP distribution in the Golgi apparatus of HeLa cells stained for p115 (cis-Golgi, red) and TGN46 (TGN, blue) at various times. (**A**) 0 time, 20 min chase and 40 min chase. At 0 time, there is little, if any correspondence between tsO45G-GFP distribution and a Golgi marker while at 20 min there is extensive correspondence in either Control or Rab6 knockdown cells, and at 40 min chase, Control cells exhibit little to no correspondence in TGN46 distribution and tsO45-GFP. In Rab6 knockdown cells, most of the tsO45G-GFP corresponded in distribution to TGN46 (blue). (**B**) Immunoblot demonstrating the high level of Rab6 protein knockdown with siRNA treatment. (**C**) Immunofluorescence evidence for high level Ra6 protein knockdown across the HeLa cell population. Software used includes iVision 4.5 (https://www.biovis.com/), Huygens Professional 4.3.0 p3 (https://www.svi.nl) and Photoshop 2019 (https://adobe.com).

Rab6 loss-of-function results in a post-ER exit delay in anterograde cargo transport, e.g., VSV-G protein, from the ER to the cell surface in which the initial steps of ER to Golgi transport appear to be normal^17,18^. Based on the literature, much of the delay could be due to post-Golgi effects; others have shown previously that Rab6 regulates post-Golgi vesicle transport through an effect on kinesin recruitment^19^. To test whether there is a significant intra-Golgi delay in cargo transport in Rab6-depleted cells, we investigated initially the qualitative effects of Rab6 knockdown on transit for a model cargo protein, i.e., tsO45-GFP, through the Golgi apparatus. In these experiments, we used siRNA directed against coding sequences shared between Rab6A and Rab6A′ and hence both isoforms were depleted equally. Temperature was used to control tsO45G-GFP release from the ER with the protein being ER retained at 39.5 °C and released from the ER at 32 °C. As shown in Fig. 1 (ortho slice view of confocal image stack, see other Figures for XY view), accumulation of tsO45G-GFP in p115-, a cis Golgi marker, positive structures were indistinguishable for Control and Rab6 siRNA treated HeLa cells; after a 20-min time chase, tsO45G-GFP co-localized with cis-Golgi marker p115 in both control and Rab6 KD cells. At late chase times (40 min), however, tsO45G-GFP in Control cells showed no correspondence with the Golgi exit marker, TGN46, while tsO45G-GFP in Rab6-depleted cells showed extensive correspondence with the TGN marker. We conclude that there is a significant intra-Golgi delay in cargo transport when Rab6 is depleted.

### Validation of a confocal line scan approach to mapping intra-Golgi cargo protein distribution

We used a confocal line scan approach^22^ to determine quantitatively the distribution of Golgi marker proteins relative to the model cargo protein, tsO45G-GFP. Work in this laboratory suggests that Rab6 plays a major role in Golgi cisternal organization. At the electron microscopy level, when Rab6 is depleted in HeLa cells, several Golgi stacks combine together to form a continuous, longer Golgi ribbon with additional cisternae and one or two dilated cisternae at trans side of the Golgi stack^18,21^ (Fig. S-1b, present work). However, when cells were gently fixed with paraformaldehyde as done for immunofluorescence analysis and then high pressure frozen, we found that the Golgi cisternae in both control and Rab6 depleted cells were dilated (Fig. S-1). Quantitatively, the distance across the Golgi stack was expanded by a factor of 2–3, a factor sufficient to make the quantitative analysis of distribution of a cargo marker, tsO45G-GFP, relative to resident Golgi proteins resolvable by deconvolved, spinning disk confocal microscopy as shown below.

**Figure 2:**
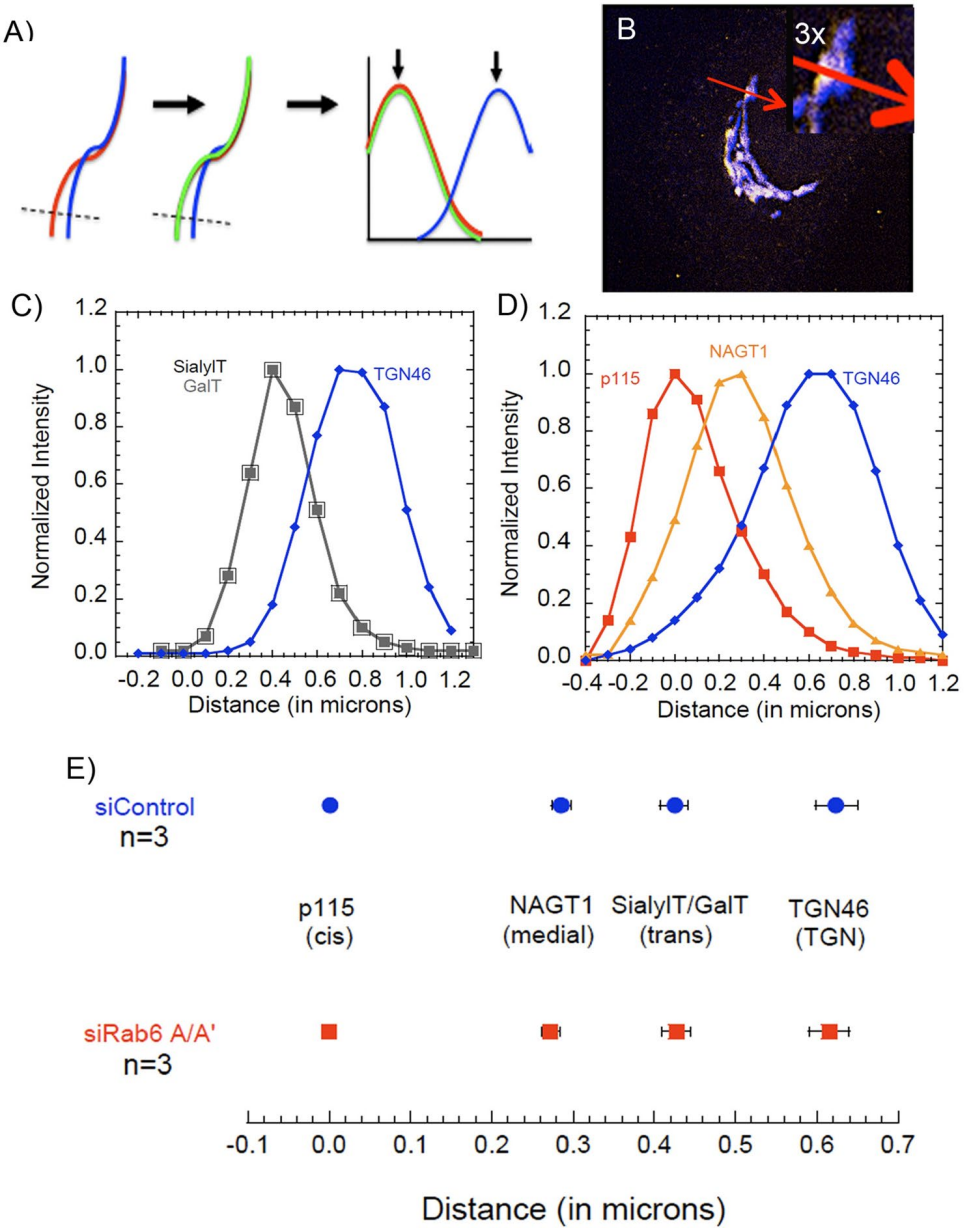
Confocal line scanning measurement of the distance between VSV-G and Golgi markers. (**A**) Schematic depiction of confocal line scanning method. In brief, after acquiring confocal image stacks, maximum intensity projections (MIPs) or the best single plane image was used for line scanning. Using ImageJ software, a perpendicular line is drawn through the Golgi ribbon in areas of maximal separation of known Golgi markers. This allows for consistent comparison amongst all data derived from this project. Pixel data is converted to distance in microns, which gives the distance in microns between your cargo marker of interest and known Golgi markers, in this instance trans marker (red) and TGN marker (blue). (**B**) An example image of an immunofluorescently labeled HeLa cell, Golgi apparatus taken with a 63X/1.40 numerical aperture objective with a CARVII spinning disk accessory and deconvolved using Huygens Professional software. The inset in the upper right-hand corner is a ×3 image blowup. (**C**,**D**) Line scan graphs as an illustrative example of the outcomes of the approach. Data are averaged for 30 or more individual line scans please see Methods for full detail. Sets of 3 different cis (p115), medial (NAGT1), trans (GalT and SialylT), and TGN (TGN46) Golgi markers are shown. (**E**) Dot plot representation of resolved Golgi markers from C and D plus and minus standard error of the mean. Distances are similar to those reported by Dejgaard et al. (2008), the originators of the line scan approach. Note that these distances are about twice that seen by electron microscopy. By electron microscopy, the Golgi cisternae in these experiments are dilated (data not shown). This is due to the weak preparative technique (formaldehyde, etc.) used to preserve antigenicity for immunofluorescence. Software used includes iVision 4.5, Huygens Professional 4.3.0 p3, KaleidaGraph 4.5.2, Excel for Mac 16.16.21 and Photoshop 2019. URLs are given in the legends for Table 1 and Fig. 1.

HeLa cells were fixed at various chase times and imaged in three color channels with two being Golgi markers. Image stacks were taken at near full cell depth, deconvolved to further sharpen the images, and then analyzed by plotting the intensity distribution of each channel along lines drawn perpendicular to the Golgi ribbon in areas of maximal separation between the Golgi markers, i.e., optimal orientation of the ribbon in 3D space (for details, see Methods). In Fig. 2a,b, this approach is illustrated for HeLa cell labeled for two trans Golgi markers, GalT and SialylT, and the TGN marker, TGN46. In the raw Golgi images, the distributions of GalT (green) and SialylT (red) are highly similar and in the merged XY image the two overlap (yellow color). XY position data were then converted to distance in microns to determine the physical distance between markers for Control and Rab6 knockdown cells (Fig. 2c–e). In total, we used 5 reference markers, p115 (cis), NAGT-1 (medial, myc-tagged stable transfectant^23^), GalT/SialylT (trans) and TGN46 (TGN). The position of the cis marker, p115, was arbitrarily set to “0”. The measured distance across the Golgi apparatus, cis to TGN, was about 0.6 µm. This distance is the same as that reported by Dejgaard et al.^22^ and approximately two to three times that determined by electron microscopy^18^. We attribute this greater distance to the point spread function of light microscopy and to cisternal dilation due to the comparatively weak fixation methods used for immunofluorescence microscopy (Supplemental Fig. 1). We note that the final endogenous protein mapped, Rab6, behaves as a trans Golgi protein (Supplemental Fig. 2). At the resolution of deconvolved, spinning disk, confocal microscopy, the distribution of the five reference markers was unaltered by Rab6 depletion (Fig. 2e).

**Figure 3:**
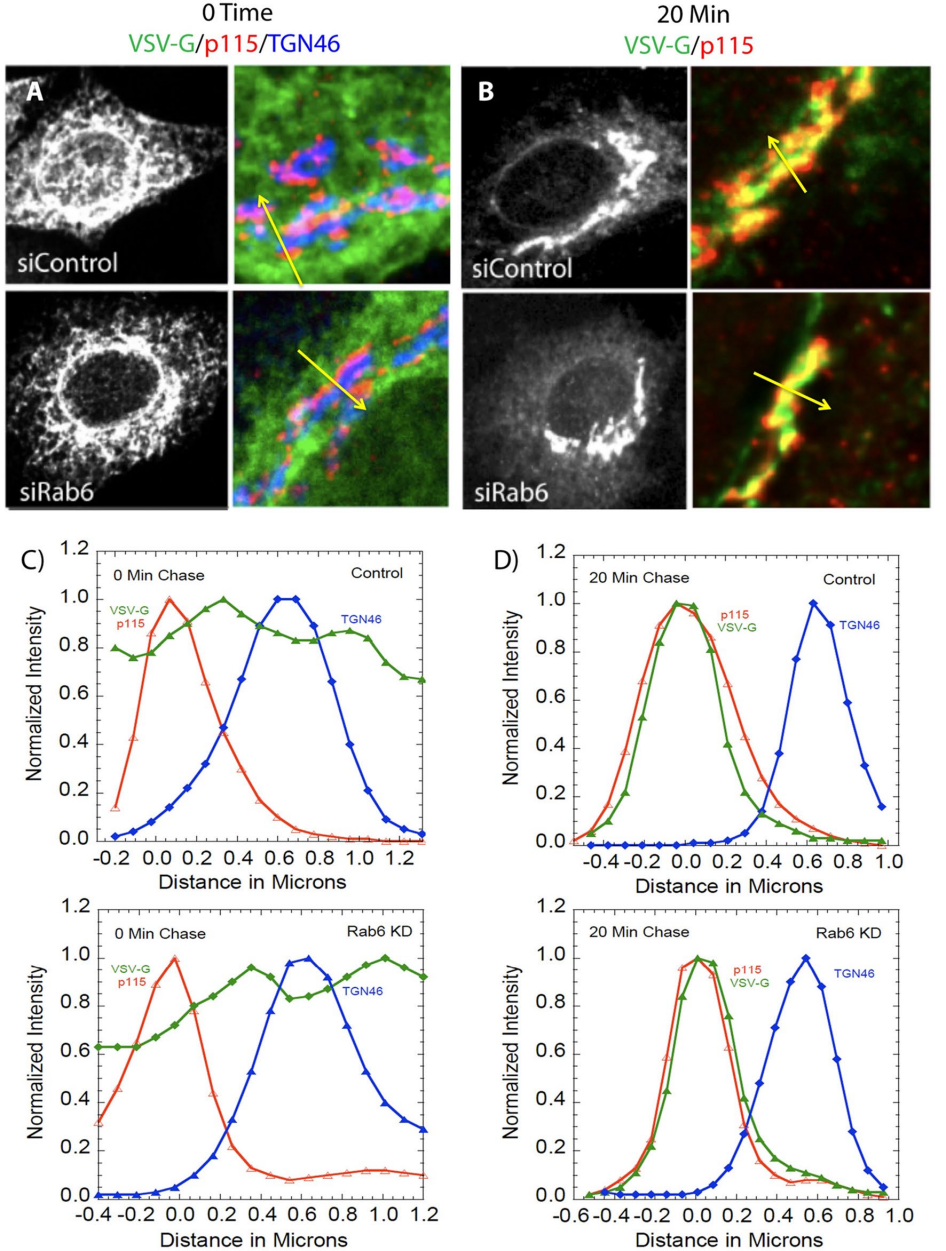
VSV-G transport kinetics from the ER to the cis-Golgi are unaffected by Rab6 depletion. (**A**) Confocal images of tsO45G-GFP at 0 min in both Control and Rab6 KD cells. At this time point, VSV-G (green) is located in the ER, as evidenced by the web-like distribution of fluorescence across the cytoplasm. There is also no correlation of VSV-G with any known Golgi markers (cis marker p115 in red, TGN marker TGN46 in blue). (**B**) Upon shifting the temperature to 32 °C, permissive temperature for transport, tsO45G reaches the Golgi at the same time in Control and Rab6 KD cells, co-localizing with cis marker p115. (**C**,**D**) Corresponding line scan graphs for 0 and 20 min time chase of VSV-G. Yellow lines trace typical line-scan-area choices. Note: The authors chose in this figure, Part A to show all 3 colors, i.e., all three markers, to illustrate visually their separation. The authors chose in this figure, Part B to show only 2 markers, 2 colors, in order to illustrate visually that with the 20 min chase the high degree of overlap between VSV-GFP distribution and that of p115. Software used includes iVision 4.5, Huygens Professional 4.3.0 p3, KaleidaGraph 4.5.2, Excel for Mac 16.16.21 and Photoshop 2019. URLs are given in the legends for Table 1 and Fig. 1.

To validate further the application of a confocal line scan approach to analyzing the intra-Golgi transport of tsO45G-GFP, we compared quantitatively the distribution of tsO4G-GFP to that of p115 and TGN46 at 0 time and 20 min chase, 32 °C, in Control and Rab6-depleted cells. As shown in Fig. 3, at 0 time, tsO45G-GFP protein displayed a web-like cytoplasmic distribution and rim-like staining of the nuclear envelope in both Control and Rab6-depleted cells, exactly the staining distribution expected for ER localization. Quantitatively, the reference Golgi proteins were well separated from one another and there was a time-independent tendency for the tsO45G-GFP distribution to peak with either of the Golgi markers. In striking contrast at 20 min chase, the relative distribution of the two cis and TGN markers was unaltered while that of tsO45G-GFP now corresponded almost exactly with that of p115 in both Control and Rab6-depleted cells. We conclude that the confocal line scan accurately reflects the distribution of tsO45G-GFP protein within the HeLa cell Golgi apparatus and that Rab6-depletion has little to no effect on initial steps in anterograde cargo transport.

### tsO45G-GFP transport stalls between the medial- and trans-Golgi in Rab6 depleted cells

**Figure 4:**
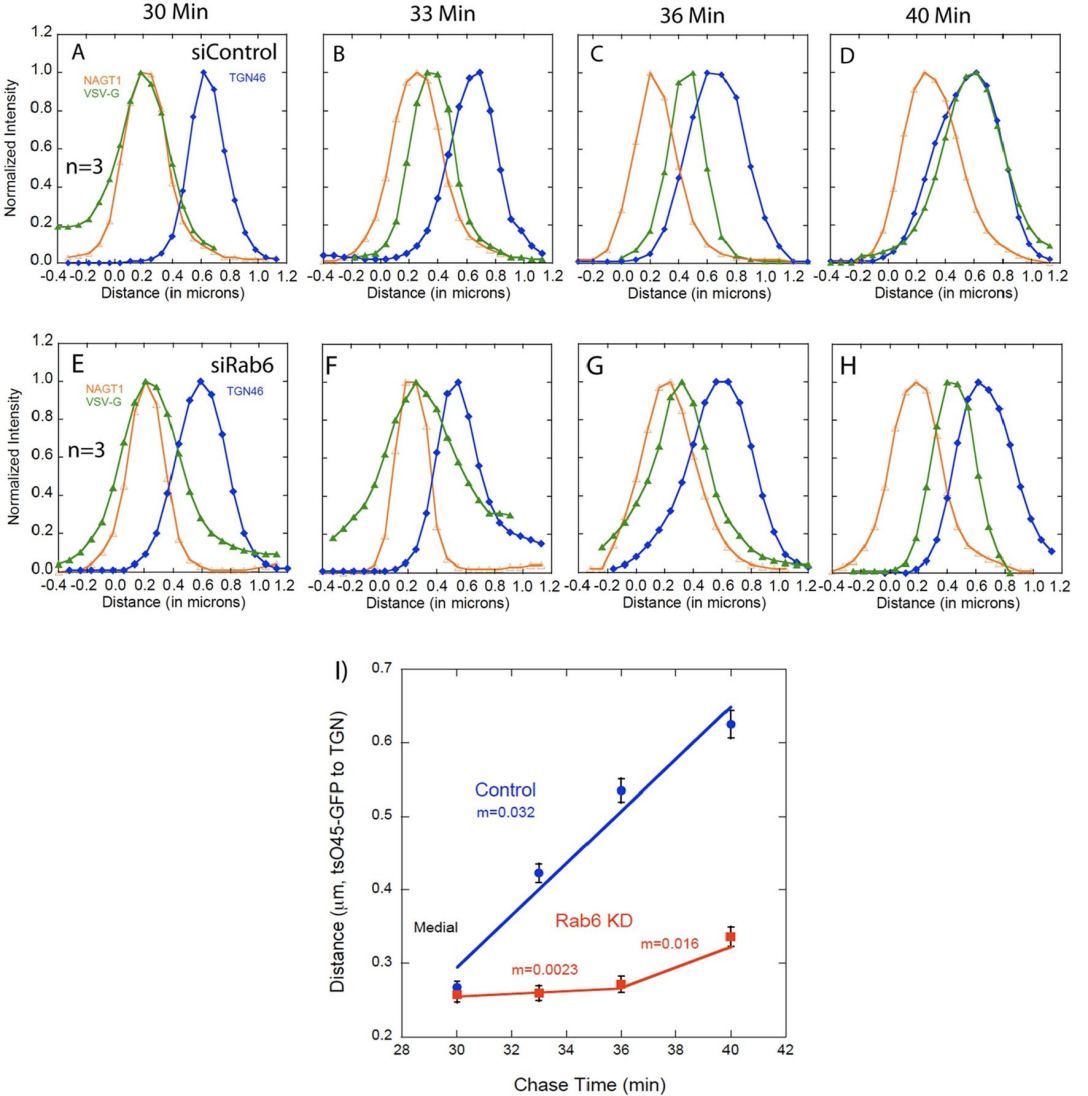
tsO45G-GFP accumulated in the medial Golgi (NAGT1 marker, 30 min chase) is slow to reach the TGN (TGN46 marker) in Rab6 KD cells. (**A**–**D**) Confocal line scan graphs depicting the medial exit kinetics of VSV-G in Control cells over time. (**E**–**H**) Confocal line scan graphs depicting the medial exit kinetics of tsO45G in Rab6 KD cells. There was a significant delay in tsO45G reaching the TGN in Rab6 KD cells. I) Quantification of medial exit kinetics in Control and Rab6 KD cells. Overall, the transport of tsO45G from the ER to the cis-Golgi or medial-Golgi was the same with Rab6 depletion. However, transport of tsO45G was slowed significantly during exit from the medial-Golgi compartment and in its transport to the TGN compartment in Rab6 KD cells, an indication that Rab6 is essential for proper intra-Golgi cargo transport at the medial-to-trans Golgi interface. Software used includes iVision 4.5, Huygens Professional 4.3.0 p3, KaleidaGraph 4.5.2, and Excel for Mac 16.16.21. URLs are given in the legends for Table 1 and Fig. 1.

**Figure 5:**
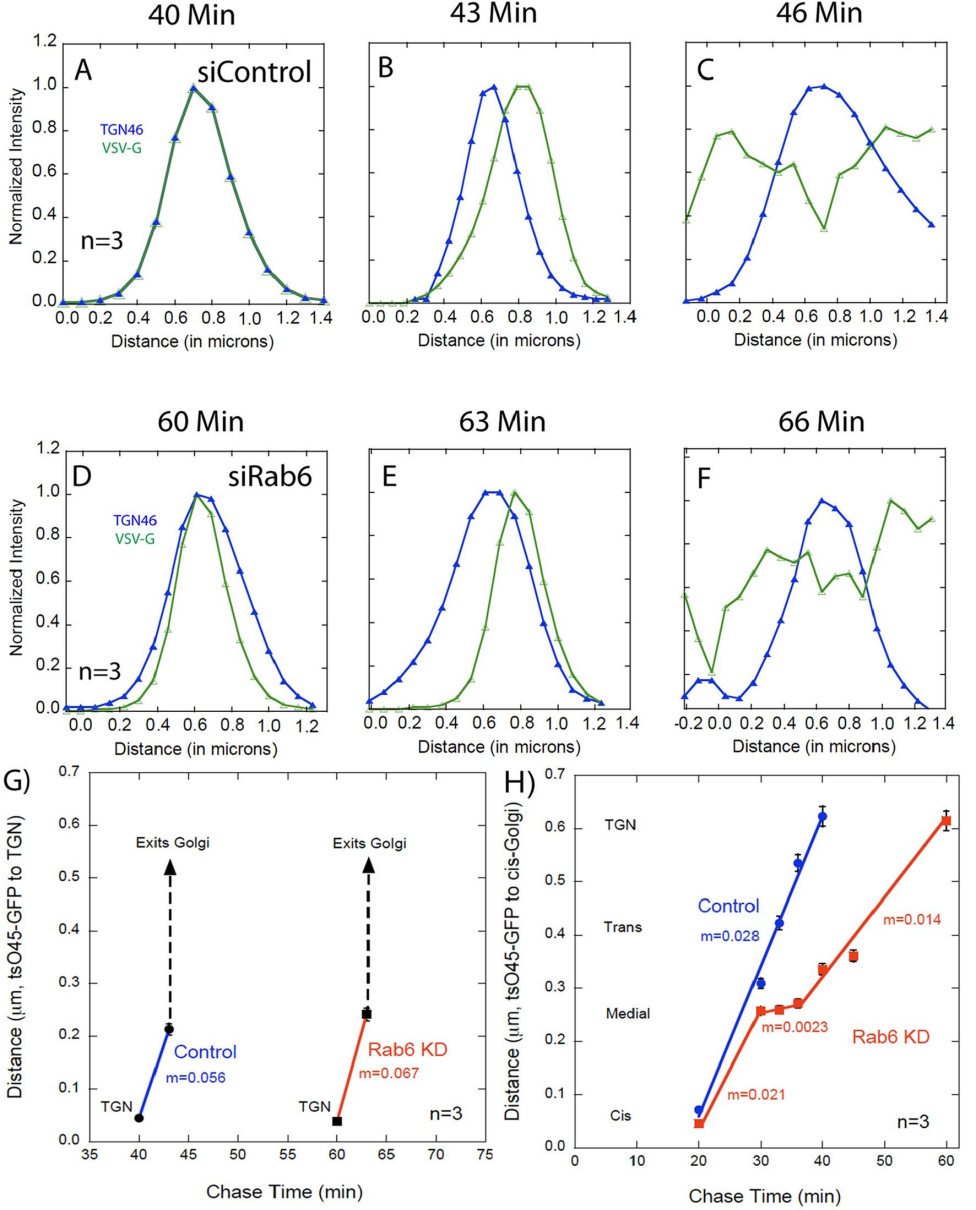
The TGN exit kinetics for tsO45G were the same in both Control and Rab6 KD cells. (**A**–**C**) Confocal line scan graphs depicting the TGN exit kinetics of tsO45G-GFP in Control cells at indicated time points. (**D**–**F**) Deconvolved, confocal line scan graphs depicting the TGN exit kinetics of tsO45G-GFP in Rab6 KD cells. While the entry into the TGN compartment was significantly delayed by 20 min in Rab6 KD, the rate of exit from the TGN was the same in Control and Rab6 KD cells. (**G**) Quantification of TGN exit kinetics in Control and Rab6 KD cells. (**H**) Overall transport kinetics of tsO45G in Control and Rab6 KD cells. Software used included iVision 4.5, Huygens Professional 4.3.0 p3, KaleidaGraph 4.5.2, and Excel for Mac 16.16. URLs are given in the legends for Table 1 and Fig. 1.

Next, we tested the hypothesis that Rab6 is preferentially required for anterograde cargo transport between the medial-Golgi and TGN. We reasoned that Rab6 as a trans Golgi Rab protein would when depleted produce phenotypic, cargo transport effects downstream of the normal trans localization of Rab6 within the Golgi apparatus, i.e., a downstream traffic jam. As shown in Fig. 4a versus e, Rab6 depletion had no effect on tsO45G-GFP transport from the cis- to medial-Golgi (compare tsO45G-GFP distribution in Figs. 3d and 4a versus e; tsO45G-GFP accumulated essentially completely with the medial-Golgi, NAGT-1 marker by 30 min chase irrespective of Rab6 status. However, as hypothesized, Rab6 depletion resulted in a strong inhibition of tsO45G-GFP transport from the medial-Golgi to the TGN (Fig. 4a–d versus e–h). In Control cells, tsO45G-GFP co-profiled with TGN46 at the end of a 40-min chase while in Rab6 depleted cells there was little, if any, accumulation of the cargo protein at the TGN. In fact, transport from the medial-Golgi to TGN was very slow initially (Fig. 4i) and full co-profiling of tsO45G-GFP with the trans markers, GalT and SialylT required more than a 45-min chase (Fig. 5). As a final, line scan, kinetic determination, we compared the TGN exit kinetics for tsO45G-GFP in Control and Rab6-depleted cells. As shown in Fig. 5g and Table 1, we found that the kinetics were virtually identical, ≤ 6 min, after allowing for a 20-min delay in cargo accumulation in the TGN with Rab6-depletion. In sum, as summarized in Fig. 5f and Table 1, there was a significant, cisternal-specific delay in cargo transport between the medial- and trans-Golgi in Rab6-depleted cells. Similar results were found with two HeLa cell variants (Fig. S-3) and two different siRNAs directed against Rab6 (Fig. S-4). We also performed a box/whisker graphical depiction of a data set (Fig. S-5). This methodology provides an alternate illustration of a time specific inhibition of cargo transport.

**Table 1:** Effect of Rab6 knockdown on the rate of tsO45G-GFP transport through the Golgi apparatus (32 °C).

Compartment	Control		Rab6 KD	
	Raw rate (nm/min)	Relative rate	Raw rate (nm/min)	Relative rate
Cis to Medial	28	1.0	21	0.75
Medial Exit	28	1.0	2.3*	0.08
Trans to TGN	28	1.0	14	0.50
TGN Exit	56	2.0	67	2.39

*p < 0.001. Assignments of Golgi regions were based on the quantitative mapping shown in Fig. 2E. Rates were calculated from Fig. 5G, H data. Based upon the line scan of the micrographs, fluorescence peaks were assigned distance value. Rates were then then determined from the slope of the respective line segment in Fig. 5H and expressed as rates in Table 1. Software used includes iVision 4.5 (https://www.biovis.com/), KaleidaGraph 4.5.2 (https://www.synergy.com/wordpress_650164087/), and Excel for Mac 16.16.21 (https://www.microsoft.com/en-us/microsoft-365/microsoft-office).

**Figure 6:**
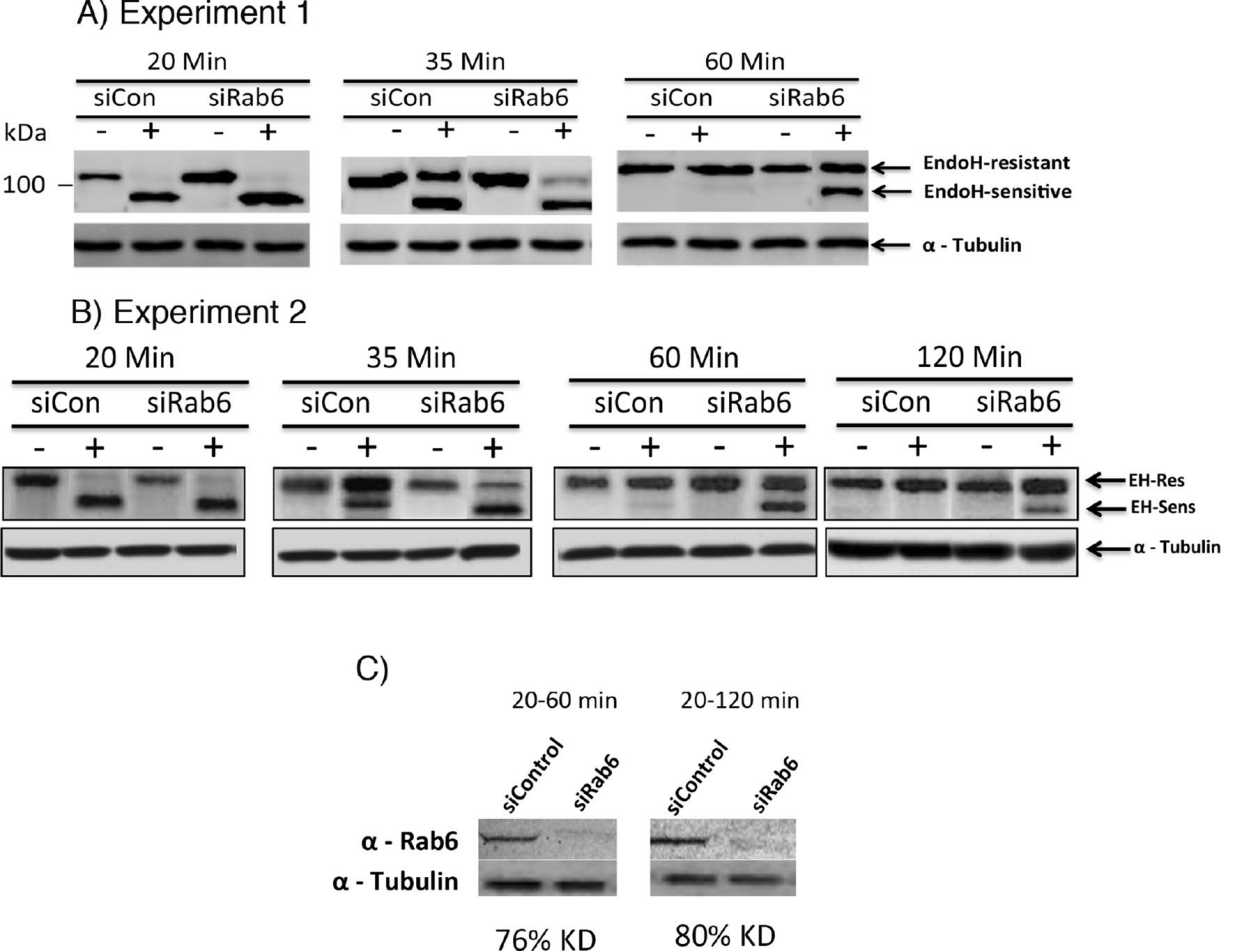
Rab6 depletion results in slow and incomplete tsO45G-GFP endoglycosidase H resistance. Control and Rab6 depleted HeLa cells were lysed at various chase times and samples incubated with and without EndoH. Following gel electrophoresis, samples were immunoblotted for GFP. (**A**,**B**) Immunoblot patterns. Note that ihe onset of EndoH resistance appeared to be the same for both Control and Rab6 depleted samples. (**C**) Rab6 protein knockdown levels by immunoblotting. Preliminary, near full length gel patterns are shown in Supplemental Fig. S-7. Software used includes iVision 4.5, Excel for Mac 16.16.21 and Photoshop 2019. URLs are given in the legends for Table 1 and Fig. 1.

To provide a non-imaging approach, we assayed for effect of Rab6 depletion on the conversion of tsO45G protein to an endoglycosidase H (EndoH) resistant form. This N-linked oligosaccharide processing step occurs in the medial-Golgi apparatus with mannosidase II removal of the last 2 mannose residues outside the N-glycan core. As shown in Fig. 6, at a 20-min chase time, tsO45G protein was fully sensitive to EndoH digestion in both Control and Rab6 knockdown HeLa cells. Over a 35 min or a 60-min chase, the protein in the case of Control cells became progressively resistant to EndoH digestion. However, in Rab6 depleted cells, tsO45G processing was much slower with a small fraction of the protein resistant at a 35 min chase and only ~ half of the protein resistant at 60 min. Even at a 120 min chase time, when in essence all the protein is cell surface, there was still a significant portion of EndoH sensitive tsO45G protein in the Rab depleted cells (Fig. 6b, c). In separate experiments and consistent with these results, siRab6 knockdown HeLa cells displayed a significant increase in GSII cell surface lectin staining (Fig. S-6). GSII as a lectin binds to exposed *N*-acetylglucosamine residues.

### Rab6 depletion inhibits intra-Golgi transport of VSVG-GFP and GPI anchored proteins at 37 °C

**Figure 7:**
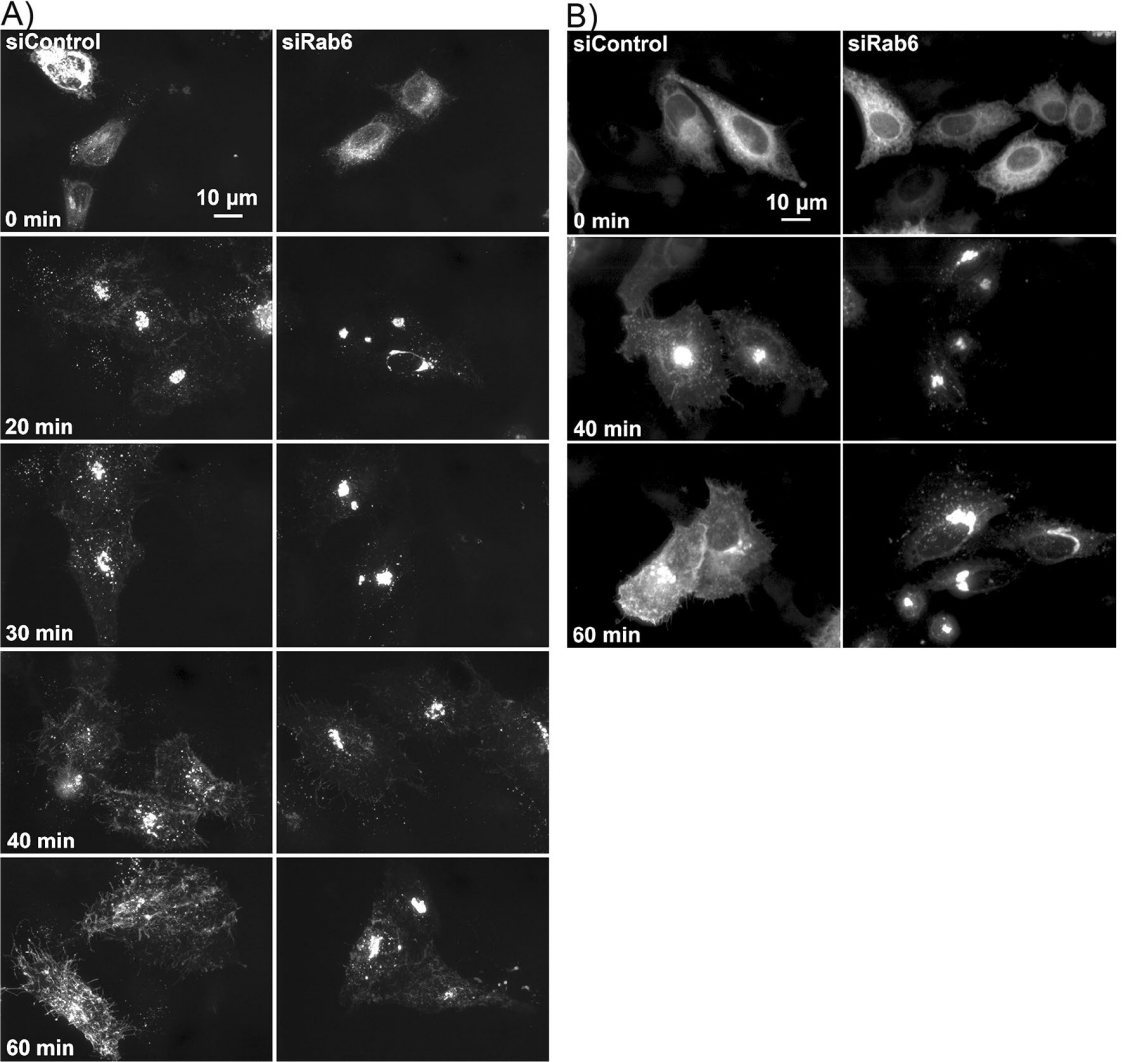
Rab6 depletion significantly delayed 37 °C cargo transport from Golgi apparatus to plasma membrane. Control and Rab6 depleted HeLa cells transfected with RUSH plasmids encoding either VSVG-GFP (**A**) or GFP-GPI (**B**). Following the addition of biotin to release the VSVG-GFP from the ER, cells were incubated at 37 °C for various chase times. Cells were then fixed and visualized by confocal light microscopy. (**A**) At 0 min, VSVG-GFP was distributed uniformly in the ER. With Rab6 depletion, the transport of VSVG-GFP from ER to Golgi apparatus showed little to no change, while the cargo transport within the Golgi apparatus was significantly delayed at 37 °C. Images shown are maximum intensity projections. (**B**) Control and Rab6 depleted HeLa cells transfected with RUSH plasmids encoding GFP-GPI were treated with biotin to release the expressed protein from the ER and then incubated at 37 °C for various chase. Cells were fixed and visualized by confocal light microscopy. At 0 min, GFP-GPI was accumulated in the ER. At the end of a 40- or 60-min chase, transport of GFP-GPI within the Golgi apparatus was slower in Rab6 depleted cells than Control cells. Images shown are maximum intensity projections. Software used included iVision 4.5 and Photoshop 2019. URLs are given in the legends for Table 1 and Fig. 1.

**Figure 8:**
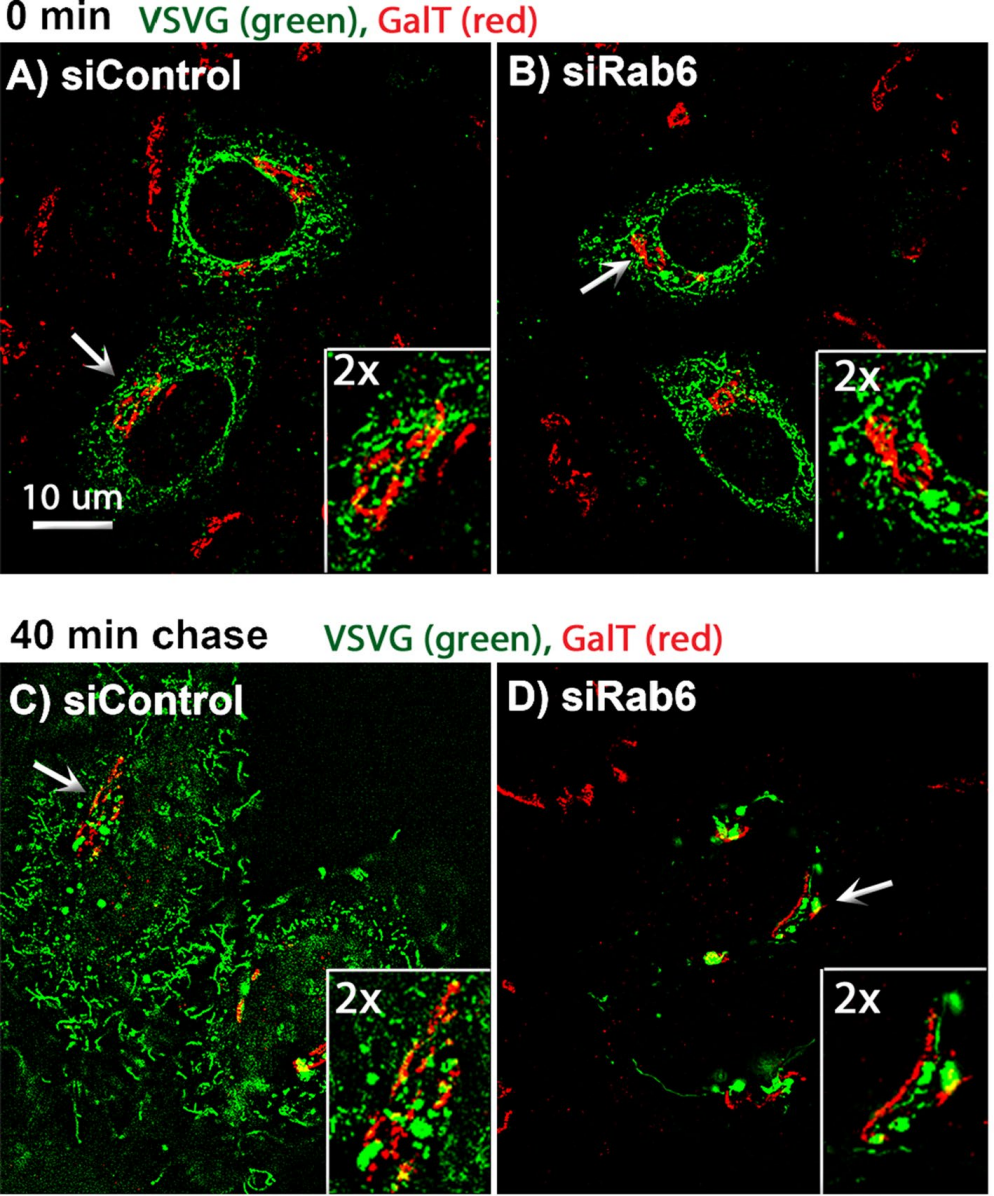
Super-resolution, 3D-SIM microscopy indicates that transport of RUSH VSVG-GFP in Rab6 depleted cells was stalled before *trans* Golgi. Control and Rab6 depleted HeLa cells transfected with RUSH plasmids encoding VSVG-GFP were incubated at 37 °C for various chase. Cells were then fixed and stained for GalT (red), and visualized by super-resolution, 3D-SIM microscopy. (**A**,**B**) At 0 min, RUSH VSVG-GFP was accumulated in the ER in both Control cells and Rab6 depleted cells. (**C**,**D**) At 40 min, in Control cells, VSVG-GFP was transported to the cells surface, while in Rab6 depleted cells, VSVG-GFP was still distributed in the Golgi apparatus and had little to no overlap with GalT distribution. Images shown are maximum intensity projections. Software used included. Software used includes Zeiss Zen 2.0 (https://www.zeiss.com/microscopy/int/products/microscope-software/zen.html) and Photoshop 2019 (https://adobe.com).

To further validate the Rab6 requirement for rapid intra-Golgi transport, we used RUSH constructs expressing VSVG-GFP or GPI-GFP to test the transport of cargo proteins at 37 °C. As shown in Fig. 7a, before treating with biotin, VSVG-GFP was distributed in the ER in both control and Rab6 knockdown cells. After addition of biotin, VSVG-GFP was released from the ER resident hook protein and started to move from ER to Golgi apparatus and plasma membrane^22^. The kinetics of VSVG-GFP transport from ER to the juxtanuclear Golgi apparatus was rapid while the transport of VSVG-GFP from Golgi apparatus to plasma membrane was significantly delayed in Rab6 depleted cells. The clearance of a second RUSH cargo protein, GFP-GPI from Golgi apparatus to plasma membrane was also slower in Rab6 depleted cells than control cells (Fig. 7b). The above results were confirmed using super-resolution, 3D-SIM microscopy. At 0 min, RUSH VSVG-GFP was accumulated in the ER in both control cells and Rab6 knockdown cells. At the end of 40-min chase, surface accumulation of VSVG-GFP was observed in control cells, while in Rab6 knockdown cells, VSVG-GFP was still distributed in the Golgi apparatus and showed at this time point little to no overlap with the distribution of GalT (red), indicative of RUSH VSVG-GFP transport that was stalled before *trans*-Golgi (Fig. 8). These results provide additional evidence for a cisternal-specific involvement of Rab6 in rapid intra-Golgi transport.

### Rab6A and Rab6A′ collectively support Intra-Golgi anterograde cargo transport

**Figure 9:**
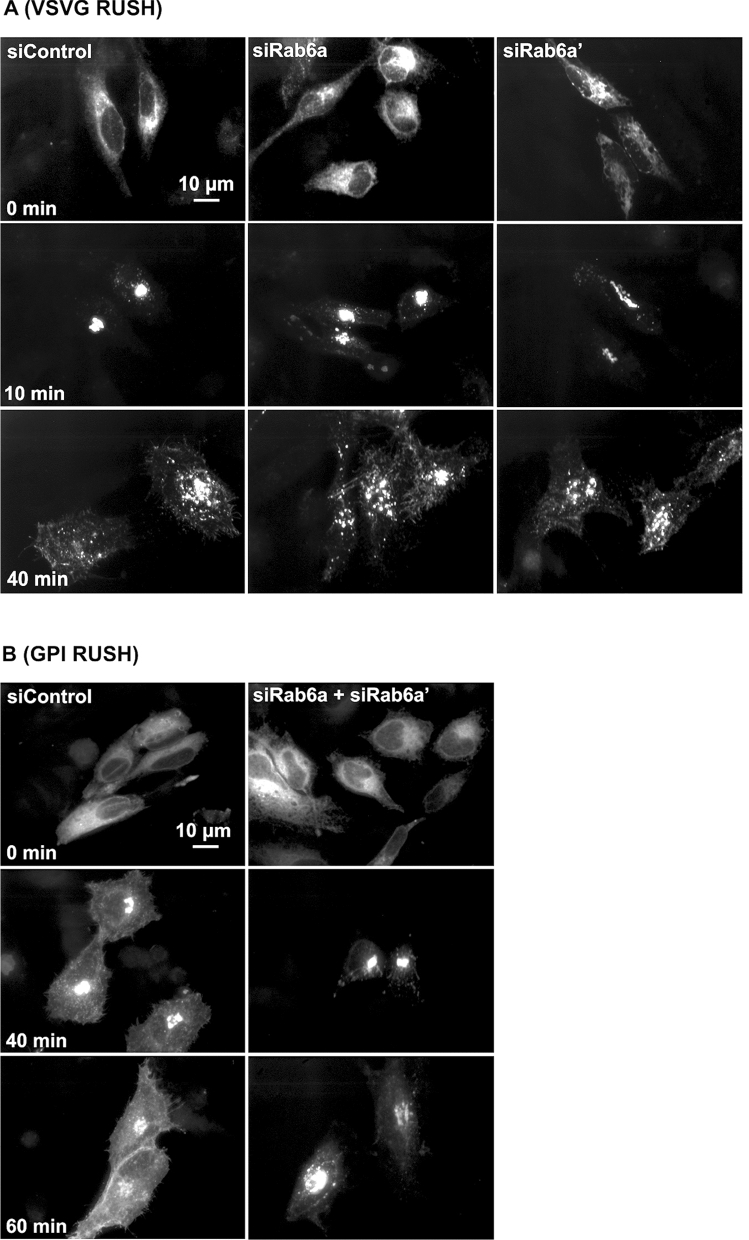
Neither siRNAs directed against Rab6A nor siRab6A′ individually inhibit anterograde cargo transport. (**A**) WT Hela were treated with siRNAs directed selectively against Rab6A or Rab6A′. Biotin was used to release at 37 °C ER accumulated Neither had any obvious effect on the transport of VSVG RUSH construct as a marker for anterograde cargo relative to Control. (**B**) Co-transfection of WT HeLa cells with siRab6A and siRab6A′, GFP-GPI RUSH cargo, strong inhibition of cargo transport. Software used includes iVision 4.5 (https://biovis.com) and Photoshop 2019 (https://adobe.com).

Previous work in our laboratory showed that individual depletion of Rab6A or Rab6A′ by selective siRNA treatment produced Golgi cisternal elongation and vesicle accumulation, albeit at half the level of a Rab6A/A′ knockdown and significantly with little-to-no increase in Golgi cisternae number^21^. Based on these results, we hypothesized that the Rab6A or Rab6A′ individually would have little to no effect on RUSH VSVG-GFP or GFP-GPI transport. As shown in Fig. 9a, the transport of VSVG-GFP from ER to the Golgi apparatus and plasma membrane was little changed with Rab6A or Rab6A′ knockdown. At 40 min, VSVG-GFP was primarily in post-Golgi vesicles. However, with co-depletion of Rab6A and Rab6A′, the intra-Golgi cargo transport was delayed. As shown in Fig. 9b, at 0 min, GFP-GPI was accumulated in the ER. At the end of a 40-min or 60-min chase, Golgi and significant surface accumulation of GFP-GPI was observed in Control cells, while GFP-GPI retention in the Golgi apparatus of Rab6A and Rab6A′ co-depleted cells was high. These results indicate that both Rab6A and Rab6A′ contribute to regulate intra-Golgi transport. Whether that contribution is by distinct individual roles or a result of mass action requires further experimentation.

## Discussion

Important roles for Rab GTPases in Golgi apparatus structure/function have been apparent for almost 3 decades with Rab6, the most abundant mammalian Golgi associated Rab protein^5^, being a particularly striking and challenging example. Rab6 has multiple effectors, at least 15; multiple roles in Golgi associated membrane trafficking; a role in Golgi associated Rab cascades; and is a determinant of Golgi structure^2–4^. A ying/yang relationship between gain-of-function and loss-of-function phenotypes make Rab6 a rare case among the roughly 20 Golgi associated Rab proteins. Overexpression of Rab6 redistributes Golgi resident proteins into the ER^13,14^ while Rab6 depletion results in delayed anterograde transport and stabilization of Golgi structure with the generation of extended Golgi cisternal stacks and increased cisternal number^17,18,20^. Bhave et al.^24^ have suggested that increased cisternal persistence could generate increased cisternal number. However, whether Rab6 regulates anterograde cargo transport through the Golgi apparatus in a local, cisternal-specific manner consistent with increased cisternal persistence, i.e., traffic jam consequences, was unknown before our studies. Our analysis demonstrated that the intra-Golgi transport of both tsO45G-GFP (32 °C) and RUSH VSVG-GFP or RUSH GFP-GPI (37 °C) were delayed within the Golgi apparatus. Under permissive conditions, all were transported normally from ER to Golgi apparatus. Detailed line scan analysis of deconvolved, confocal images containing cis, medial, trans and TGN markers indicated normal kinetics for tsO45G-GFP transport from cis to medial Golgi and normal TGN exit kinetics. Strikingly, medial Golgi exit kinetics were slowed more than tenfold. Similar line scan results were obtained using 2 different validated Rab6 siRNAs. siRNAs directed specifically against Rab6A or Rab6A′ had no effect on cargo transport despite substantial protein knockdown. Under these conditions, there is no increase in Golgi cisternal number^21^. Biochemical analyses revealed altered functionality as indicated by time-correlated acquisition of EndoH resistance of tsO45G. Furthermore, by 3D-SIM analysis, a super resolution technique, the delay in RUSH VSVG-GFP transport was found to be pre-trans Golgi apparatus. In brief, multiple analytical approaches demonstrate that Rab6 is required for rapid, intra-Golgi, cargo transport between the medial and trans-Golgi cisternae, a step immediately downstream of the normal trans-Golgi localization of the protein and the site of Rab6-dependent vesicle accumulation^18^.

These results support a model for Rab6(A/A′) functionality in which anterograde cargo trafficking phenotype is separated spatially from the normal intra-Golgi apparatus distribution of the Rab6A/A′ proteins, the two very closely related Rab6 isoforms. Multiple lines of evidence show Rab6 to be a trans-Golgi protein^8,22^ (present work). Here, by line scan, deconvolved confocal, immunofluorescence analysis, we demonstrated Rab6 to coincide with the two trans-Golgi marker proteins, GalT and SialylT. As might be expect, Rab6-dependent, Golgi vesicle accumulation is also trans-Golgi specific^18^. Considering these different factors, how then can trans Golgi protein(s) be determinant for events that happen in the medial Golgi apparatus? One appealing possibility is that the outcome is the consequence of slowed cisternal maturation leading to increased medial Golgi persistence time and hence increased Golgi cisternal number as postulated by Bhave et al.^24^. In this model, we place the trans Golgi Rab, Rab6, is a key determinant for the retrograde trafficking required in a cisternal maturation/ progression model of Golgi function to mature the medial Golgi into a trans Golgi cisterna. Hence a traffic jam at this interface should result in increased downstream, medial cisternal persistence time and a selective increase in Golgi cisternal number. In fact, we observed through multiple readouts in Rab6 knockdown HeLa cells a strong, local increase in intra-Golgi transit time. Our proposed, detailed extension of the Bhave et al. model is supported by our additional data that knockdown of neither Rab6A nor Rab6A′ alone was sufficient to affect anterograde cargo transport and as shown recently neither affected Golgi cisternal number^21^. Further evidence consistent with this model comes from prior data dating back to the late 1990s that Rab6 is a key determinant of retrograde Golgi trafficking^15^. Alternatively, these outcomes could be explained by a model in which Rab6 can act directly at the medial Golgi by shuttling back and forth between the medial and trans Golgi with a set of rate constants in which the Rab appears to be a trans Golgi resident protein. In support of this model, one might take as an example mannose 6-phosphate receptors which bind ligand within the Golgi apparatus but appear at steady-state to be late endosomal/multivesicular body resident proteins. In a shuttle model, one is still left with the problem of explaining the apparent strong linkage between anterograde transport rate and Golgi cisternal number. We favor a cisternal persistence model. Rab43 provides another recent example of a Rab protein that affects intra-Golgi anterograde cargo transport events at the medial Golgi level^25,26^. In this case, the primary effect is proposed to be upon the routing of cargo proteins to different subregions within a Golgi cisternal compartment. Here routing was shown to be dependent on the sequence of the cytoplasmic domain of the transmembrane cargo protein. In brief, we give weight to the increased cisternal number observed in Rab6 knockdown HeLa cells as a basis for favoring a cisternal maturation/progression model versus another Golgi models^27–33^.

In conclusion, we present evidence that Rab6, a trans-Golgi protein, is a key determinant of intra-Golgi, anterograde cargo transport rate at the level of medial-to-trans Golgi transport. Our data indicate an increase in medial Golgi persistence time and as such suggest a delayed cisternal progression explanation for the observed increase in Golgi cisternal number in Rab6 depleted HeLa cells^18,21^. To the best of our knowledge, these are the first data linking a Rab protein to downstream events in intra-Golgi anterograde cargo transport and concomitant regulation of Golgi cisternal number.

## Methods

### Antibodies and lectins

The antibodies and lectins used in the work were purchased from commercial suppliers or were generous gifts from individual researchers. These included antibodies against the following antigens: p115 (mouse monoclonal, BD Technologies, 1:100), 9E10 (mouse monoclonal, Santa Cruz, 1:100), TGN46 (sheep polyclonal, AbD Serotech, 1:100), SialylT (goat polyclonal, Santa Cruz, 1:200), GFP (rabbit polyclonal, Thermo Fisher, 1:100), Rab6 (rabbit polyclonal, Santa Cruz, 1:100), GalT (rabbit polyclonal, gift^34^). The lectin GSII (Alexa Fluor 488 conjugate, Thermo Fisher, 1:100) was used to stain *N*-acetylglucosamine residues in fixed cells.

### Cell culture

Wild type and NAGT1-myc HeLa cells were cultured in Dulbecco’s Modified Eagle’s Medium (DMEM) supplemented with 10% fetal bovine serum (Atlanta Biologicals) in a humidified incubator at 37 °C and 5% CO_2_^16,18,21^. For expression of tsO45G protein, HeLa cells were cultured at 39.5 °C to allow accumulation of protein in the ER overnight, and then transferred to media that has been prewarmed to 32 °C, conditions permissive for anterograde transport of tsO45G to the cell surface^18^.

### RNA interference

siRNA directed against Rab6, Rab6A and Rab6A′ have been described previously^12,16^. All siRNAs used for this work were manufactured by Dharmacon RNA Technologies, a division of Thermo Fisher. siRNAs were transfected at a final concentration of 100 nM, using Oligofectamine Transfection Reagent (Invitrogen) in the absence of FBS according to previously described protocols. In brief, for visual analysis, 70,000 cells were seeded per 35 mm tissue culture dish containing 12 mm diameter coverslips (Fisher Scientific) one day prior to siRNA transfection (Day 0). Successive cycles of siRNA transfections were performed on Day 1 and Day 2. For light microscopy experiments, cells were fixed with 3.4% paraformaldehyde or cold methanol (− 20 °C) 96 h (4 days) post initial transfection. For Western blotting, cell lysates were prepared from individual tissue culture dishes for subsequent quantitative assessment of protein depletion^16^.

### Electron microscope (EM) analysis of Golgi organization

HeLa cells were cultured on sapphire discs as previously described^21^ and transfected with Control or siRNA directed against Rab6 as above. 96 h post-transfection, cells were either immediately processed for high-pressure freezing (HPF) and freeze substitution (FS) as previously described^21^ or were paraformaldehyde fixed, saponin permeabilized per the immunofluorescence procedure described below prior to subsequent HPF/FS and EM. All EM was done as described^21^.

### VSVG transfection and chase

WT and NAGT1 HeLa cells (70,000) were seeded on coverslips in 35 mm dishes 1 day prior to siRNA transfections. Transfections were carried out as previously described^16^, however on Day 4 after incubations with either siControl or siRab6 A/A′, HeLa cells were transfected with plasmids encoding GFP-tagged tsO45 mutant VSVG protein using FuGENE HD transfection reagent (Promega). Posttransfection cells were incubated overnight at 39.5 °C, a nonpermissive temperature for VSVG transport from the ER to the Golgi apparatus. To chase, cells were shifted to 32 °C, permissive temperature for VSVG transport, in the presence of cycloheximide to prevent further protein synthesis^18^. Cells were incubated at 32 °C for various chase times, then fixed with 3.4% formaldehyde and stained with various Golgi markers. Quantitative results are the mean of three independent experiments.

### RUSH expression and chase

Wild type HeLa cells were treated with the corresponding siRNA as described above. Typically, 48 h after the first transfection cycle, cells were transfected with RUSH plasmid encoding VSVG-GFP or GPI-GFP using FuGENE HD transfection reagent (Promega) according to the manufacturer’s protocol. Expression of RUSH constructs in HeLa cells and chase were performed as described previously with minor modifications^35^ (plasmids were a gift of Franck Perez, Institut Curie). In brief, transfected cells were incubated overnight at 37 °C. 1 × 10^−7^ M avidin (Sigma-Aldrich) was added for efficient retention of cargo proteins in the ER. Cells were then incubated in the presence of 40 μM biotin (Sigma-Aldrich) to release the cargo proteins. Cells were incubated at 37 °C for various chase times in the presence of cycloheximide to prevent further protein synthesis. Cells were then fixed with formaldehyde. Confocal image stacks were taken as described above.

### Fluorescence microscopy and image processing

To assess the effect of protein knockdown on Golgi phenotype, HeLa cells (wild type or stably expressing NAGT1myc) were grown on coverslips and transfected with either Control or Rab6 A/A′ siRNAs. 96 h post siRNA transfections, cells were fixed with 3.4% formaldehyde and stained with Rab6 antibody^16^. Widefield microscopy and spinning disk confocal imaging were performed with a Zeiss Axiovert 200 M microscope (Carl Zeiss) fitted with high numerical aperture (NA) objectives: 20X/0.8 NA, 63X/1.40 NA and 100X/1.40 NA and a CARV II spinning disk confocal accessory (BD Bioimaging) mounted to the side port of the microscope^16^. The microscope was controlled with iVision Mac (BioVision). For the analysis of VSV-G fluorescence distribution relative to known cis, medial and trans Golgi markers (see below), images were first deconvolved using Huygens Professional 4.3.0p3 software to sharpen the confocal images. Reported results are the average across 30 or more cells per condition and time point. Line scan analysis of VSV-G distribution relative to Golgi markers was done as described below.

### Confocal line scan analysis and microscopy

The distributions of several Golgi proteins, including cis localized p115, medial localized NAGT1, trans localized GalT and SialylT, trans Golgi network localized TGN46, and VSV-G-GFP were analyzed from peak intensities along a line track drawn perpendicular to the Golgi ribbon. In brief, ribbon-like Golgi apparatus structures in a plane giving the brightest Golgi staining and maximal separation of Golgi markers were chosen (5–7 areas per Golgi, ≥ 30 cells per condition). The pixel intensities along the line track for these markers were plotted versus distance in microns using ImageJ software and the distances between peak intensities were determined for each time point. Spinning disk confocal image stacks for Golgi apparatus visualization were collected through the entire depth of the cell and either compressed into a single plane using maximum intensity projection (MIP), or as in most cases the best single plane was chosen for confocal line scanning. Images were color joined using iVision Mac software. Deconvolution to sharpen further the confocal fluorescent images was done using Huygens software (SVI). Line scanning was done in the ImageJ freeware program, and images were prepared for publication with Adobe Photoshop and Illustrator software (Adobe Systems).

### 3D-SIM

3D-SIM microscopy was performed with Zeiss ELYRA PS.1 super-resolution system. Image stacks were collected with 100x/1.46 oil objective and processed with Zen software (Zeiss). The images were taken at the same exposure time. The image stacks were compressed into a single plane using “3D” processing mode in Zen software.

### SDS-PAGE and western blot analysis

SDS-PAGE and western blotting were performed to determine knockdown of protein level for each experiment using commercial antibodies and reagents. Protein samples were analyzed by electrophoresis on either 8% or 10% polyacrylamide gels followed by transfer to nitrocellulose membrane. The blots were incubated first with primary antibodies, and then with a secondary IgG antibody conjugated with IRDye 800 (light sensitive). The blots were scanned and analyzed with an Odyssey Infrared Imaging System (LICOR Biosciences).

### Endoglycosidase (EndoH) assays

Control and siRab6 treated HeLa cells transfected with tsO45G-GFP for 18 h at 39.5ºC were shifted to permissive temperature, 32ºC, for various times. Cells were detergent lysed with 1% Triton X-100 in isotonic buffer as described by Whitt et al.^25^. Lysates were denatured, made acidic with citrate buffer following the manufacturer’s instructions (New England BioLabs), and incubated in the presence or absence of EndoH prior to immunoblotting with anti- GFP antibodies.

### Statistics

Unless otherwise noted in the figure legend, experiments were repeated 3 times and data points are reported as means and error bars were calculated as the standard deviation of the mean.

## Supplementary information

Supplementary information
