# Supplementary material for: Rab6 is required for rapid, cisternal-specific, intra-Golgi cargo transport
Source: Sci Rep. 2020 Oct 6;10:16604. doi: 10.1038/s41598-020-73276-w (PMC7538953; doi:10.1038/s41598-020-73276-w)
Supplement: Supplementary file 1 — Supplementary information [file 41598_2020_73276_MOESM1_ESM.pdf]

Supplemental Figures

Rab6 Is Required for Rapid, Cisternal-Specific, Intra-Golgi Cargo Transport

Lindsey James Dickson[#], Shijie Liu[#], and Brian Storrie*

Department of Physiology and Biophysics

University of Arkansas for Medical Sciences

Little Rock, Arkansas 72205

[#]Co-first authors

*Corresponding Author

Supplemental Figures

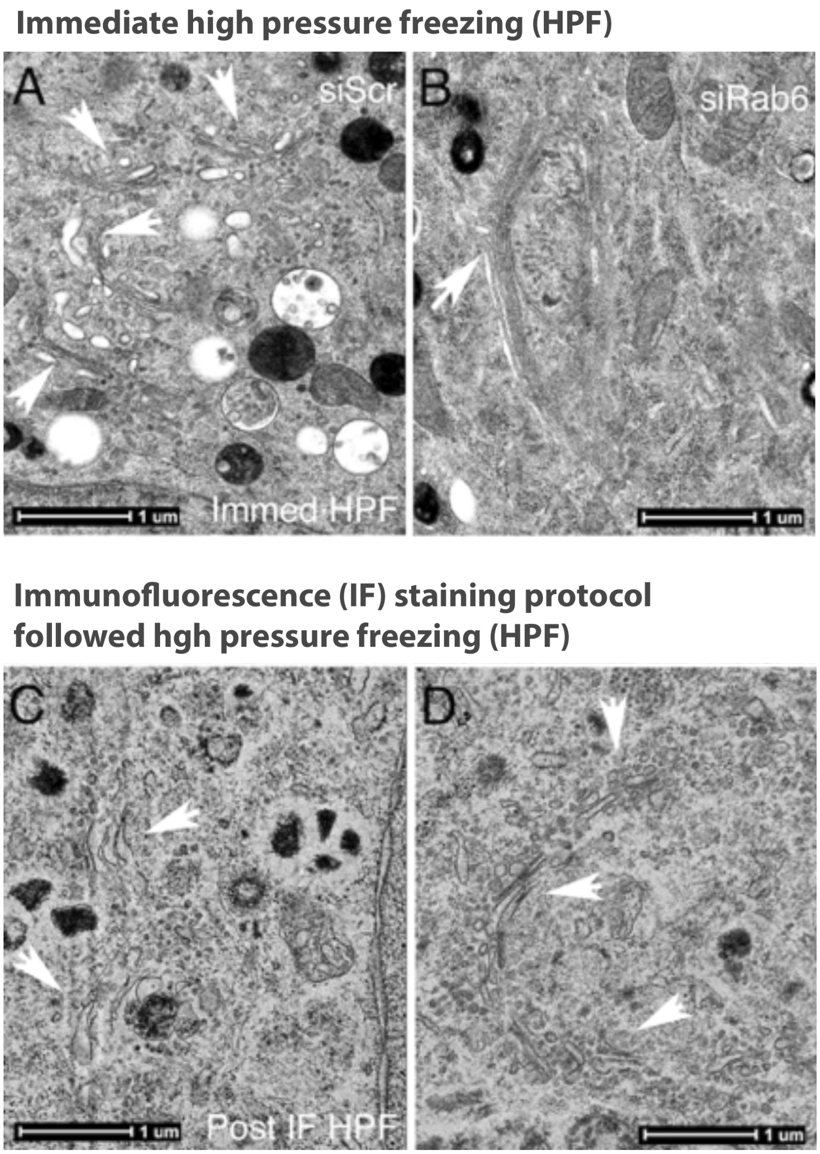

Figure S-1. Immediate HPF/FS preparation of samples for EM yields a compart spacing of Golgi cisternae while delaying HPF/FS processing until immununofluorescence (IF) processing yields a dilated Golgi apparatus . A) Immediate HPF/FS processing of siScr (Control) treated HeLa cells, Golgi rich region of cell. B) Immediate HPF/FS processing of siRab6 treated HeLa cells, Golgi rich region of cell. C) Post IF procedure HPF/FS of Control HeLa cells, Golgi rich region of cello D)

Post IF procedure HPF/FS of siRab6 HeLa cells, Golgi rich region of cell. Arrowheads point to Golgi cisternal elements within Control and siRab6 treated cells.

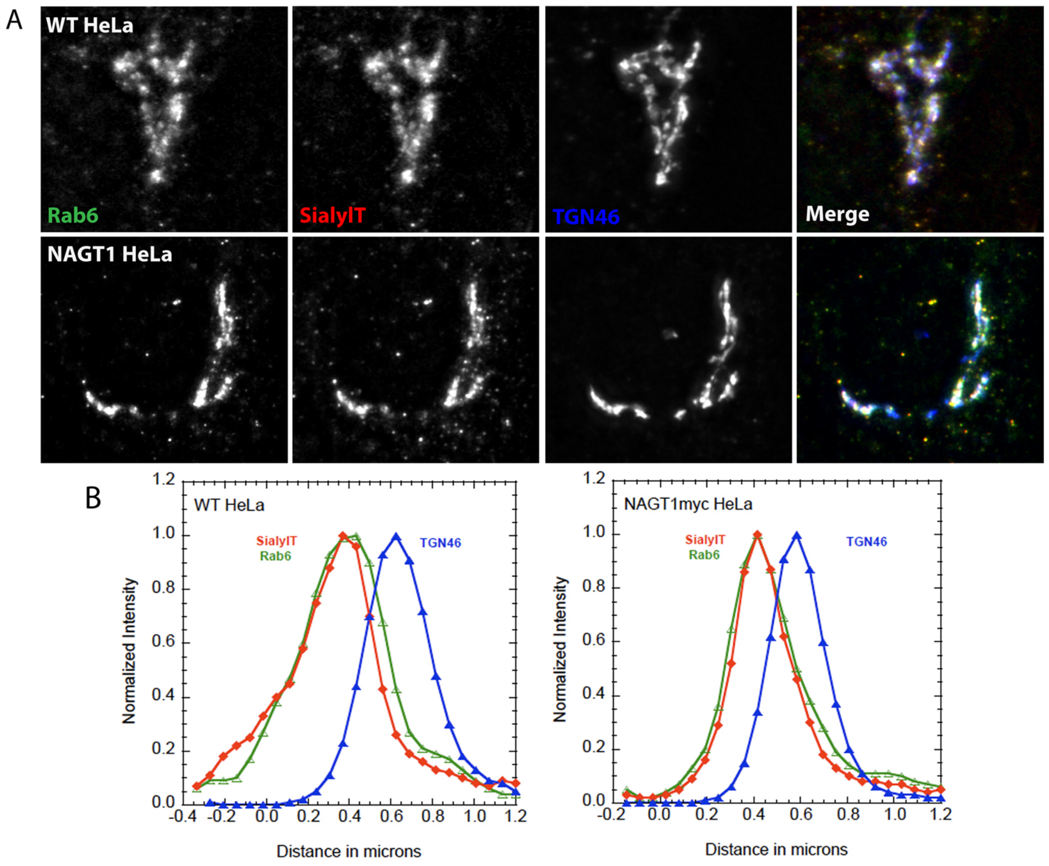

Figure S-2. Rab6 in both WT and NAGT1 HeLa cells co-distributes with the trans–Golgi marker, SialylT. A) Deconvolved, confocal Immunofluorescence images of WT or NAGT1myc HeLa cells stained for Rab6 (green), trans marker SialylT (red), and TGN46 (blue). B) Confocal line scan graphs derived from immunofluorescence images.

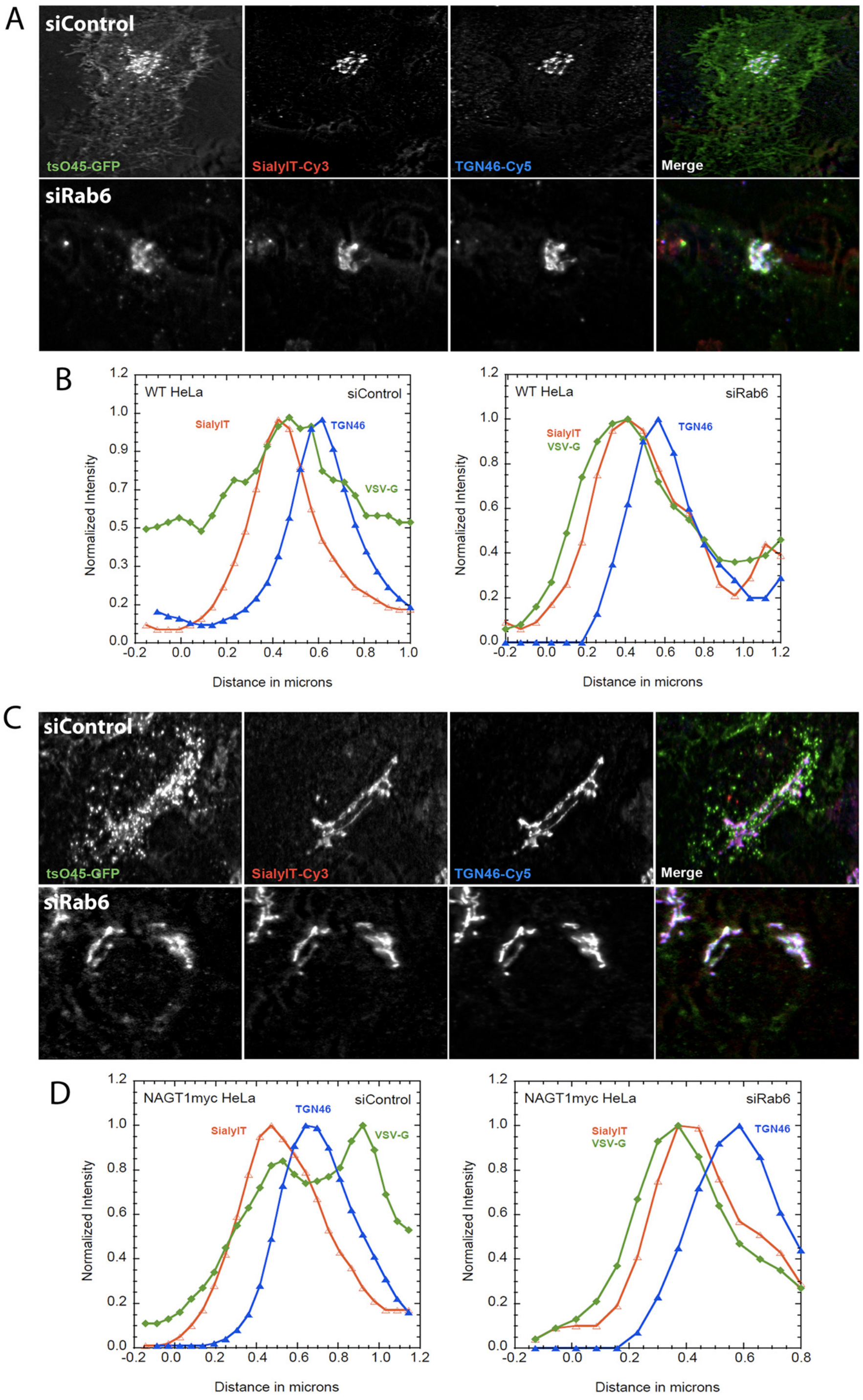

Figure S-3. tsO45-GFP (VSVG) at 45 min chase time distributes similarly in both WT and NAGT1 HeLa cells. A) Deconvolved, confocal images of tsO45G distribution at 45 min chase time in both Control and Rab6 KD WT HeLa cells. At this time point,

siControl tsO45-G (green) is mainly located at the plasma membrane and in post-Golgi vesicles. In siRab6 cells, tsO45G co-localizes with the trans marker, SialylT. Similar results were seen in NAGT1myc HeLa cells (B). C&D) Corresponding confocal line scan graphs for 45 min chase in both WT and NAGT1myc HeLa cells.

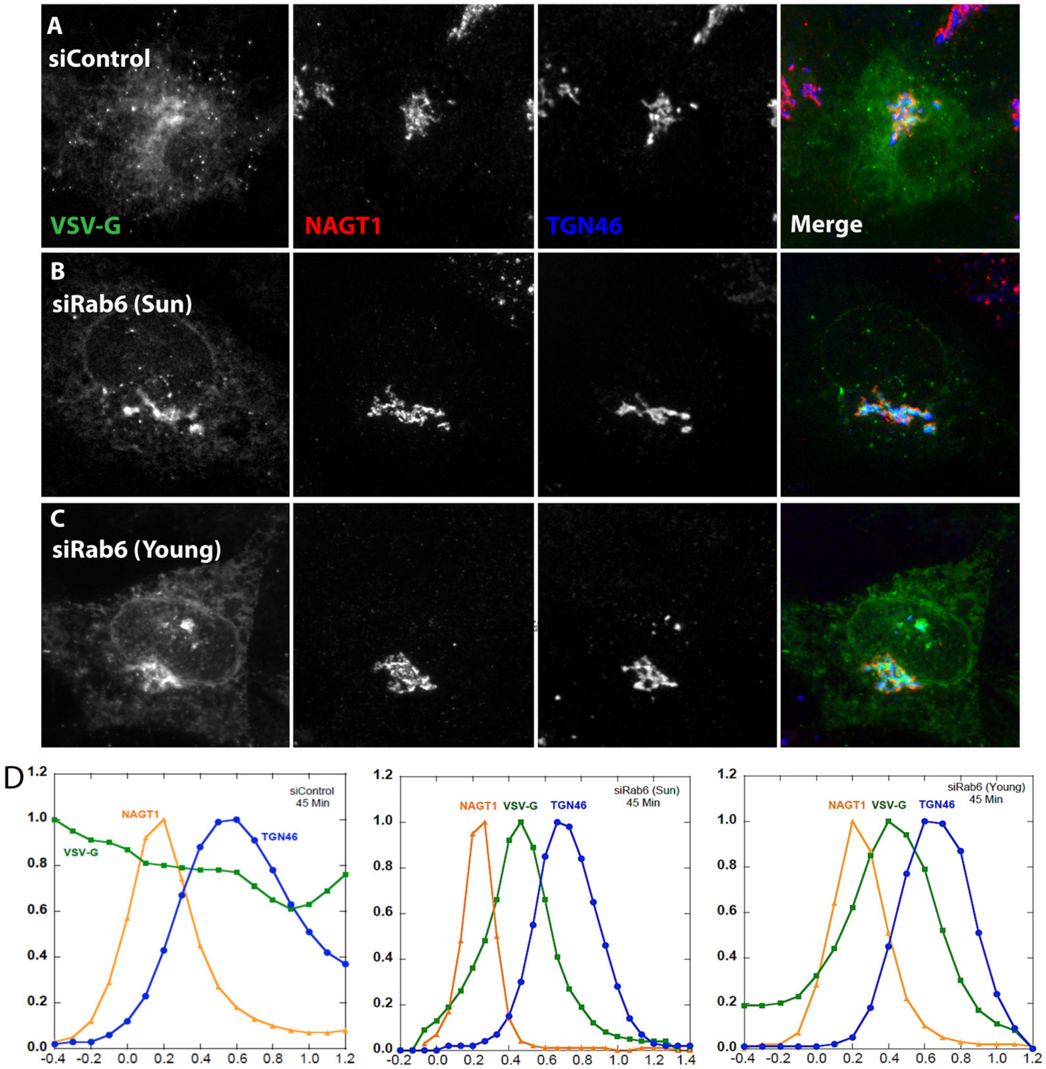

Figure S-4. Two different siRNAs directed against Rab6 gave the same delay in tsO45G (VSVG) transport.

Two different siRNAs, siRab6(Sun; Sun et al., 2007) and siRab6(Young; Young et al., 2005) were used to knockdown Rab6 in NAGT1 HeLa cells. Cells were then transfected with tsO45G-GFP and after a 45-min chase at permissive temperature cells were fixed and stained for NAGT1 and TGN46. A-C) Deconvolved, confocal immunofluorescence images of tsO45G-GFP (VSVG), NAGT1 (NAGT1-myc) and TGN46 distributions in Control (A), siRab6(Sun)-treated cells (B), and siRab6(Young)-treated cells (C). D) Confocal line scan graphs of corresponding images.

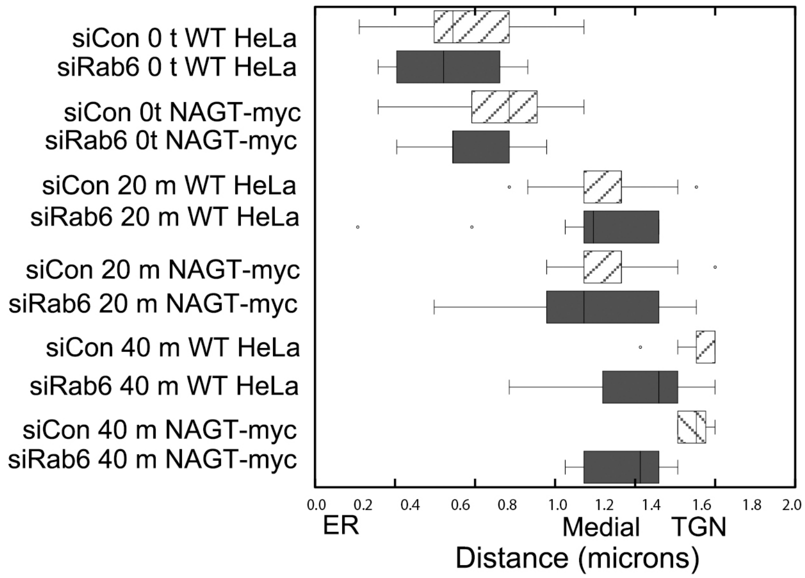

Figure S-5. Rab6 depletion has no effect on tsO45G-GFP transport from ER to the cis-Golgi or medial-Golgi. But tsO45G-GFP transport was slowed significantly during exit from the medial-Golgi compartment in Rab6 KD cells. Open slashed boxes siControl, filled boxes siRab6 VSV-G location; Times: 0, 20, 40 min chase; Experiments done in WT HeLa and in NAGT-myc stable HeLa transfectant.

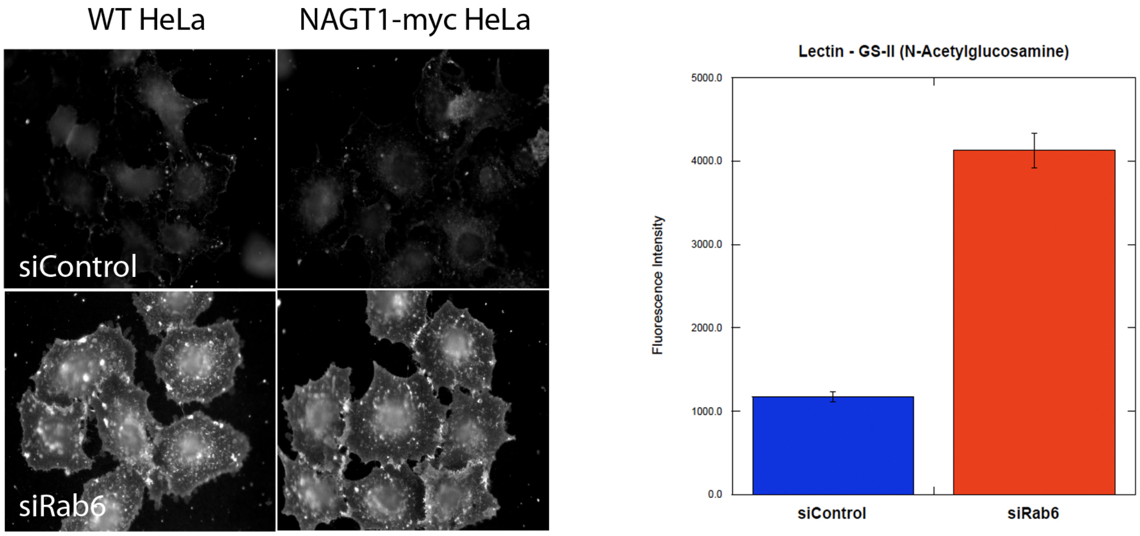

Figure S-6. GSII cell surface staining example data for WT HeLa (left hand micrographs) and NAGT1-myc HeLa (stable transfection, right hand micrograph). In the siControl treated cells (top row), the level of cell surface GSII staining was low. In the siRab6 treated HeLa cells (bottom row), the level of cell surface GSII staining was decidedly higher in both cases. Intensity quantification of the fluorescence levels indicated an ~3-fold increase in intensity. This result is exactly that expected for decreased medial-specific glycosylation.

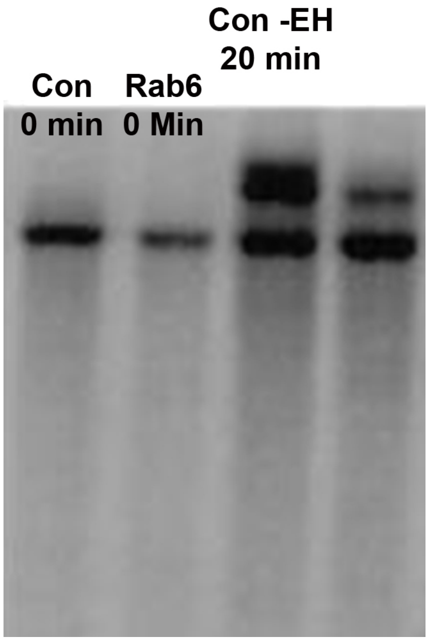

Figure S-7. Preliminary, near full length gel patterns show that Rab6 depletion results in slow and incomplete tsO45G-GFP endoglycosidase H resistance. Control and Rab6 depleted HeLa cells were lysed at various chase times and samples incubated with and without EndoH. Following gel electrophoresis, samples were immunoblotted for GFP.
